# Neighborhood effects and consequences of criminal justice contact: a research framework

**DOI:** 10.1007/s43762-024-00138-w

**Published:** 2024-10-11

**Authors:** Ling Wu, Na Li

**Affiliations:** 1 Department of Justice Studies, Prairie View A&M University, Prairie View, USA; 2 Department of Computer Science, Prairie View A&M University, Prairie View, USA

**Keywords:** Federal Statistical Research Data Center, Criminal Justice Administrative Records System, Neighborhood effects, Residential mobility, Socioeconomic inequality

## Abstract

This paper proposes a framework to examine how neighborhood factors influence criminal justice (CJ) contact and contribute to disparities across multiple stages of the justice process. By conceptualizing the punishment process as a dynamic set of decision-making points, this study highlights the role of neighborhood context in shaping offenders’ CJ trajectories and post-CJ residential inequality. Using Harris County, Texas, as a case study, this research considers individual-, neighborhood-, and event-level variables to understand the cumulative effects of neighborhood characteristics on CJ outcomes. This study underscores the critical need to investigate neighborhood mobility and its broader implications for community development and public policy. The findings can be supported by extensive data from the Federal Statistical Research Data Centers and the Criminal Justice Administrative Records System, offering a robust analysis of offenders’ spatial patterns and economic transitions.

## Introduction

Urban researchers and criminal justice scholars have long been interested in examining how neighborhood effects influence criminal justice (CJ) contact, particularly in terms of how neighborhood factors contribute to disparities at various stages of the justice process. CJ contact functions as a sorting mechanism, disproportionately affecting socially marginalized populations (Wakefield & Uggen, 2010). This mechanism is deeply intertwined with community and ecological factors, such as neighborhood disadvantage, demographic characteristics, and crime levels (Wang & Mears, 2010). Neighborhood context plays a crucial role in shaping offenders’ CJ contact and provides a framework for understanding how early contact with the justice system can set in motion cycles of inequality, leading to diverse trajectories of punishment (Tonry, 2015). For instance, residing in a disadvantaged neighborhood, along with other indicators of marginalization, can influence both the intensity and duration of CJ involvement over time (DiPrete & Eirich, 2006). A notable example is the disproportionately high incarceration rates of young male minorities from poor neighborhoods (Wooldredge, 2007). Despite the clear relevance of neighborhood characteristics, research explicitly analyzing their correlation with CJ contact is surprisingly scarce, largely because neighborhood factors are rarely captured in CJ process data (Spohn, 2000).

Extant research on inequality in the CJ system has overwhelmingly focused on the disparity in a single decision-making case, capturing only a snapshot of a very dynamic process that constitutes criminal punishment (Hagan, 1974). Studying disparities across multiple stages in the CJ system is challenging, because CJ administrative data at various stages are usually handled by different organizational providers and it is almost impossible to track offenders across the CJ stages. However, investigating neighborhood effects across successive stages of the justice system is critical. The extent that offenders from disadvantaged neighborhoods are treated more punitively is always visible or similar in each single stage (Spohn & Fornango, 2009). Alternatively, disparities that occur at one stage of the justice system may be partially or wholly offset by subsequent case-processing decisions (Kurlychek & Johnson, 2019). Without examining multiple case outcomes, it is not reliable to assess the joint and cumulative effects of neighborhood on punishment (DiPrete & Eirich, 2006).

The goal of this paper is to develop a framework for investigating the relationship between neighborhood characteristics and CJ contact. We conceptualize the punishment process as a dynamic series of decision-making points, drawing on the work of Baumer (2013) and Ulmer (2012). Using the analytical procedures from Kutateladze et al. (2014), we can estimate neighborhood disparities across multiple CJ decision points for a large sample of justice-involved individuals in a major urban area. Our framework incorporates three groups of factors: individual characteristics, neighborhood variables, and CJ event-level variables. Specifically, neighborhood-level factors—such as concentrated disadvantage, ethnic heterogeneity, and residential instability—serve as structural sources of disadvantage with persistent direct and indirect effects on various CJ outcomes (Ye & Wu, 2011; DeMarco, 2024). By examining how these neighborhood characteristics correlate with CJ contact and shape case outcomes at each stage of the CJ process, while controlling for individual and legal factors, this paper aims to illuminate the crucial role neighborhoods play in influencing CJ trajectories. This framework directly addresses the central issue of how neighborhood contexts impact CJ contact and outcomes.

Secondly, this framework aims to examine offenders’ trajectories of neighborhood attainment as consequences of CJ contact, specifically considering how the forms and levels of CJ contact may maintain and exacerbate offenders’ post-CJ contact residential inequality and concentration. CJ contact is tied to broader patterns of inequality outside of the CJ system though generating deleterious consequences over a diverse range of social and economic outcomes long after system contact has expired. It serves as a key turning point that alters one’s life trajectory because having a criminal record can create an array of lifelong obstacles to employment, housing, and many others that stand in the way of successful return to a normative lifestyle (Apel & Sweeten, 2010). In addition, CJ contact has a broad policy implication for communities and society. CJ population, as a large subset of general population in U.S. (one in three Americans have some type of criminal record), is highly concentrated in certain neighborhoods both before and after CJ contact.

Neighborhood mobility of the CJ population is an understudied but critical factor in both individual outcomes and community development. Residential mobility after CJ contact significantly influences offenders’ life opportunities and recidivism risk (Warner, 2016). For example, research shows that downward mobility increases the likelihood of recidivism, while upward mobility reduces it (Kubrin et al., 2007). CJ contact, whether through arrest or incarceration, stigmatizes individuals, diminishes employment prospects, and often marginalizes them further (Pager et al., 2009). As a result, offenders frequently experience downward mobility, moving into poorer neighborhoods (Warner, 2016), in contrast to the general population, which tends to follow upward mobility trends (Warner & Remster, 2021). Despite these findings, the mechanisms that drive justice-involved individuals into particular neighborhoods remain unclear (Lee et al., 2022). This research framework aims to investigate how the level of CJ contact shapes offenders’ mobility patterns, particularly through the spatial and economic transitions of residential mobility (Li et al., 2021).

Offenders’ mobility trajectories, while unique to individuals, often exhibit spatial patterns at the neighborhood level, which can have adverse effects on communities (Wu et al., 2015). CJ populations are highly concentrated geographically, with certain neighborhoods experiencing a higher proportion of residents being removed (transition from community to custody) or reentering (transition from custody to community) (Leipnik et al., 2016). For instance, in Chicago, the removal rate in predominantly Black communities is much higher than in white communities (Sampson, 2012). Similarly, reentry patterns show that more than half of a sample of parolees resettled in less than 10% of Chicago neighborhoods (Visher & Farrell, 2005). These removal and reentry patterns generate not only spatial concentration but also spatial mismatch, as offenders do not necessarily return to their original neighborhoods after reentry, leading to concentrations in different areas. Given that most offenders eventually return to communities, governments are increasingly aware of the strain these places on neighborhoods with high concentrations of CJ-involved individuals (Clear et al., 2001). These neighborhoods face resource challenges, including the need to reduce recidivism, provide employment opportunities, and stabilize the lives of returning individuals and their families, all while reducing crime and exercising social control.

To explore these patterns, this paper conceptualizes the analytics of the spatial and socioeconomic mobility of the CJ population across 786 census tracts in Harris County, Texas, as a case study. The analysis will leverage restricted data accessed through Federal Statistical Research Data Centers (RDC), including the Criminal Justice Administrative Records System (CJARS) for Harris County, TX (1978–2009), and restricted 2000 and 2010 decennial censuses. Our focal population will consist of offenders arrested in Harris County in 2001, examining their subsequent CJ contact events between 2001 and 2009, as well as their prior criminal records from 1978 to 2000. CJARS provides detailed information on offenders’ personal characteristics and CJ events, such as arrest and incarceration, while the decennial censuses offer neighborhood-level data. To identify offenders’ residential neighborhoods, we will link CJARS’ anonymized roster tables to restricted decennial census data using Protected Identification Keys (PIKs). Public versions of the 2000 and 2010 censuses, along with TIGER/Line + GIS data, will support spatial analysis. Once the census tract identifiers for offenders’ origin and destination residences are established, neighborhood variables from the public-use decennial censuses will be retrieved. We will utilize harmonized tract-level data from public-use IPUMS, which provides standardized time series data for the 2000 and 2010 censuses based on 2010 geographic units. These variables will allow us to assess neighborhood conditions before and after CJ contact. In addition, 2010 TIGER/Line + GIS data will support spatial analysis of offenders’ neighborhood attainment trajectories. Through a research framework integrating these datasets and conducting spatial analysis, this paper aims to demonstrate how CJ contact influences offenders’ residential mobility patterns and the socioeconomic conditions of the neighborhoods they move to, thus fulfilling the broader research objective of understanding the relationship between neighborhood context and CJ outcomes.

This research contributes to the Census Bureau under Title 13, Chap. 5 of the U.S. Code. It satisfies responsibilities of 13 U.S.C. § 101 by producing statistics about crime and the justice system. This research will benefit the Census Bureau by preparing a series of estimates of the CJ population and models that will measure the cumulative disadvantage with individual-, event-, and tract-level variables (Criterion 11). The geo-identifier and tract-level variables will provide the measure of trajectory of neighborhood mobility and the geographical distribution pattern of CJ population. Therefore, this research framework will improve the utility of Census Bureau data for analyzing demographic, economic, or social conditions of CJ population with many public policy applications (Criterion 3). Because CJARS is a new dataset and has not been highly used by researchers, we propose to identify how CJARS data could be linked to other data sets. The CJARS data is only accessible through an RDC, and so it will test the quality of this new criminal justice microdata and help improve or promote the data (Criterion 9).

## Data and variables

We request to access CJARS data between 1978 and 2010 in Harris County, TX. CJARS is a CJ process repository which integrates various events in the same CJ episodes associated with offenders. It is only available to qualified researchers approved by the RDCs. The time frame (1978–2010) and geographical setting (Harris County, TX) of this research are chosen to fulfill the research goal to examine the relationship between neighborhoods and CJ contact, with consideration of the coverage of the current CJARS data holding (Finlay & Mueller-Smith, 2021). We will use roster, arrest, adjudication, and incarceration tables in CJARS to select our focal cohorts and subcohorts, and access information on induvial-level variables (age, gender, race, ethnicity) and event-level variables (offence characteristics and criminal outcomes). CJARS does not have any neighborhood information of offenders. We will take advantage of CJARS’s ability to link to other demographic data sources through PIKs to identify offenders’ residences and neighborhoods. Restricted decennial censuses 2000 and 2010 will be used to identify offenders’ residence MAFIDs and census tracts. Thus, we will be able to identify specific census tracts where offenders resided in 2000 and 2010 respectively and trace their residential mobility between origin and destination census tracts. Public versions of decennial censuses 2000 and 2010 are used to retrieve neighborhood variables (concentrated disadvantage, ethnic heterogeneity, residential instability). These variables are needed to assess the conditions of neighborhoods where offenders resided before and after CJ contact. We will utilize harmonized tract data from public-use IPUMS (https://data2.nhgis.org/main) which provides geographically standardized time series tables to support high-quality tabulations of one year’s census data for another year’s geographic units. We will also use 2010 TIGER/Line + GIS data from public IPUMS NHGIS (https://data2.nhgis.org/main). NHGIS modified the TIGER/Line based on boundaries derived from the U.S. Census Bureau’s 2010 TIGER/Line files, and added a GISJOIN attribute field, which supplies standard identifiers that correspond to the GISJOIN identifiers in NHGIS data tables.

The CJARS data track each CJ episode for an offender from arrest to discharge from the justice system. A CJ episode refers to the complete chain of events as a criminal case is processed, possibly by different agencies, through the justice system (Finlay & Mueller-Smith, 2021). Arrest serves as an offender’s initial CJ contact, the first stage of a CJ episode, and the entry point into the CJ system. Each arrest’s progression is different as the case is processed through the justice system, and a case proceeding may end at a certain point before, during or after adjudication. This paper develops a framework to examine a focal CJ caseload population, offenders arrested in Harris County, TX in 2001 as the case study. The focal cohorts are selected by using booking dates from the CJARS Harris County arrest table in 2001. Then, CJARS Harris County data between 2001 and 2009 are used to identify our focal CJ events which include focal cohorts’ initial arrests and all subsequent events, such as adjudication, sentencing, and incarceration, in the same CJ episodes.

CJARS as the only integrated CJ process repository in U.S. collects various administrative data held by a wide range of federal, state, and local CJ agencies. Even though CJARS intends to ultimately build a national justice process repository, its current data holding does not warrant a national study of CJ process or even a state-wide examination of all CJ stages/domains due to its limitation on geographical, temporal, and procedural coverage. Data availability varies substantially across jurisdictions considering time frame and domain coverage, from states with no data coverage to states with state-wide coverage in two or more CJ domains within certain time frames. This paper intends to study cumulative disadvantage in the CJ system by tracking offenders from arrests to final sanctions, and hence needs to strategically define its geographical setting and time frame based on detailed geographical, temporal, and procedural coverage of the current data holding as depicted in the publicly available CJARS documentation (Finlay & Mueller-Smith, 2021).

CJARS documentation has no information on the size of CJ population or event counts in any state or county. Texas is the state with the largest incarcerated population (154,749) in U.S. and can possibly provide us a large sample size to support statistical analyses (The Sentencing Project, 2022). CJARS collects data in Texas from Texas Department of Criminal Justice, Texas Department of Public Safety, iDocket, County Clerk’s Offices and County District Clerk’s Offices in 69 counties (27% of all counties in Texas), 6 county sheriff’s offices, and one municipal police department. State-wide data coverage is available for domains like court (1993–2013) and prison (1978–2018), but arrest data only have partial coverage and can be accessed if a specific county shares such data with CJARS. Harris County, Texas, is chosen as our research setting because the CJARS arrest data from the Harris County Sheriff’s Office includes detailed booking information. As the most populous county in Texas, Harris County offers a large and diverse criminal caseload from a major urban jurisdiction.

The time frame (2001–2009) for selecting focal cohorts and CJ events is based on the temporal coverage of CJARS data in Harris County and the research goal of examining the relationship between neighborhoods and CJ contact. Even though CJARS has no neighborhood identifiers or information on neighborhood conditions pertaining to offenders, such information can be possibly accessed through linking CJARS with other data containing neighborhood variables. CJARS data scheme is designed to be able to link anonymously at the person-level to selected demographic and socioeconomic data within the Census Bureau Data Linkage Infrastructure through unique personal identifiers named Protected Identification Keys (PIKs).

Decennial censuses count every resident in the U.S. based on short forms. Restricted versions of decennial censuses are part of the Census Bureau Data Linkage Infrastructure and can be used to identify residential neighborhoods for all individuals in the U.S. who are not incapacitated on Census Day (April 1, 2000). The restricted decennial censuses 2000 and 2010 have information on individuals’ PIKs, MAFIDs (Master Address File Identification Numbers, which are unique identifiers assigned to housing unit addresses), and the finer geographical units where they reside, such as census tracts. Census tract is considered as a neighborhood generally encompassing 2,500 to 8,000 persons and can be used as the geographical unit. In the U.S., census tracts are designed to be relatively homogeneous units with respect to population characteristics, economic status, and living conditions. Because the anonymized CJARS roster table contains both PIKs and *cjars_ids* (personal identifiers assigned by the CJARS team to uniquely identify justice-involved offenders), the roster table can serve as a crosswalk between the CJARS event tables and restricted decennial census data. Linking focal cohorts in CJARS roster table and restricted decennial census 2000 allows us to identify offenders’ residences and corresponding census tracts (in 2000) before they were arrested in 2001.

This research also examines offenders’ trajectories of neighborhood attainment as consequences of CJ contact. We will trace offenders’ residential mobility from origin neighborhoods before arrests to destination neighborhoods after reentering the society. We will use a subset of the focal cohort by focusing on those offenders who, after arrests in 2001, were not incarcerated or were released from prison before the end of 2009. We can then identify these subcohorts’ origin residence census tracts in 2000 and destination census tracts in 2010, through linking these subcohorts in CJARS roster table and restricted decennial censuses 2000 and 2010. Potential data attrition occurs when situations, such as death, homelessness, or immigration, make offenders identified in decennial census 2000 untraceable in 2010.

Once census tract identifiers are known for our focal cohorts and subcohorts, variables pertaining to neighborhood conditions can be achieved through public-version decennial censuses 2000 and 2010. Geographical configurations of census tracts may change over time. From 2000 to 2010, the number of census tracts increases from 649 to 786 in Harris County. National Historical Geographic Information System (NHGIS) in Integrated Public Use Microdata Series (IPUMS) USA provides geographically standardized time series tables to support the tabulations of decennial census data of different years (e.g., 2000 census tract population within the 2010 census tract boundary). We will utilize various time series tables harmonized to 2010 census tracts from public-use IPUMS Data (https://data2.nhgis.org/main) to access tract-level data from decennial censuses 2000 and 2010. Therefore, neighborhood variables are available for the same 786 census tracts in Harris County for both 2000 and 2010. All restricted and public access data mentioned above can provide the individual-, event-, and neighborhood-level variables as summarized below and in Table 1.

### Individual-level variables

Individual characteristics, such as age, gender, race, and ethnicity, have been found to significantly shape CJ outcomes (Spohn & Fornango, 2009). CJARS data frame has a roster table which provides *cjars_id* and information on personal characteristics (date of birth, sex, race, and ethnicity) for each offender. This paper plans to use all individual variables from CJARS roster table to study the relationship between neighborhoods and CJ contact. *Age* is a continuous variable measured in years. *Sex* is a dichotomous variable with two attributes, male and female. *Race* has five categories, including White, Black, Asian or Pacific Islander, American Indian or Alaska Native, and Other. *Ethnicity* is a dichotomous variable with two attributes, Hispanic and non-Hispanic. All individual variables are considered as extra-legal offender characteristics, which are generally innate in nature but can possibly impact the decision making in the CJ system (Bankston, 1983; Spohn, 2000).

### Event-level variables

CJARS data frame includes relational tables at the event level, such as arrest and adjudication tables at the charge level, and incarceration table at the term level. *cjars_id* is available in all relational tables. Each table also has a unique event identifier for that respective type of event contained in the table, such the *arr_id* uniquely identifying arrests, *adj_id* for court filings, and *inc_id* for incarceration events. In addition, each table has an event identifier that can be used to link to the event that led to the event described in the table. For example, the adjudication table contains both *adj_id* (identify a specific case filing) and *arr_id* (identify the specific arrest leading to the specific case filing). Using *cjars_id* and all event identifiers in relational tables can integrate all events in one CJ episode for an offender, because we can not only pool specific events associated with a single offender, but also reconstruct a chain of events in one CJ episode belonging to that offender.

All event-level variables utilized in this research are derived from key original variables in three relational tables in CJARS: arrest table (the booking date, raw and standardized offense code of the arrest); adjudication table (raw charge offense description, raw and standardized offense grade, raw and standardized legal code, raw and standardized disposition offense, case disposition date, raw and standardized disposition, raw sentence, incarceration length, sentencing probation length, suspended sentence, fine amount); incarceration table (incarceration entry and exit date, raw description of facility and standardized facility type, raw description of entry type and standardized entry status, raw description of exit type and standardized exit status).

Event-level variables indicate either case characteristics or intermediate and final case outcomes. Research has suggested legally relevant factors exert the strongest influence on punishment (Mitchell, 2005; Spohn, 2000). The first group of variables consists of legally defined offender and offense characteristics. The study will only examine each offender’s “top offence”, the salient charge facing each offender, considering it is common for offenders to be charged with multiple offenses arising from the same criminal incident. Top offence is chosen as the offense assigned the most severe sentence. *Offense type* measures the top offence category for person, property, drug, driving under the influence (DUI), public order, and other offences, based on the offense classification scheme in CJARS. *Statutory severity* represents the seriousness of the top charge by using a dichotomous variable charge grade (Felony-level or Misdemeanor-level charge). *Charging characteristics* is described by the number of charges pressed against an offender (continuous variable) which can be calculated by linking focal cohorts in roster table and their charges in adjudication table. Offenders’ criminal history is an important source of disadvantage to predict downstream punishment decisions (Welch et al., 1984). Many criminal decisions, such as arrest, prosecutors’ charging, and judicial decisions are explicitly or inexplicitly tied to assessments of prior CJ contact (Kutateladze & Lawson, 2017). We will use two common measures of *criminal history*, prior arrest (presence or absence of prior arrests) and prior incarceration (presence or absence of prior incarcerations). We need to identify arrest and/or incarceration records before 2001 associated with focal cohorts. The earliest available CJARS Arrest table and Incarceration table in Harris County is in 1978. We will therefore retrospectively locate focal cohorts’ arrest and incarceration records between 1978 and 2000 by linking the focal cohorts in roster table in 2001 and arrest and adjudication tables in CJARS Harris County (1978–2000) through *cjars_id*.

The second group of variables involves the procedural factors. *Initial plea* is based on the intensity of the denial of guilt indicated, including plea of guilty and plea of not guilty. It can be identified in “raw disposition description” in the adjudication table. *Charge alteration* indicates whether the most serious initial charge facing a defendant is changed by prosecutors who decide to go forward with a prosecution but choose to deviate from initial charging decision made by police, and upcharge (downcharge) by filing more (less) serious charges against defendants. Three attributes of Charge Alteration, including charge increase, decrease, or no change, can be identified by comparing “raw offense code” in the Arrest Table and “offense charged at case filing” in the adjudication table. Both procedural factors are used to explore the relationship between charge dynamics and adjudication/sentencing outcomes for the purpose of predicting final disposition.

The third group of variables consists of decisions made by justice administrators which reflect intermediate and final case outcomes. *Case dismissal* measures whether the case is dismissed by the prosecutor or judge, based on “standardized disposition” in CJARS adjudication table. Dismissal terminates a case and becomes the final judgment of that case, and defendants whose case are not dismissed will remain in the CJ system for further processing. *Adjudication disposition* measures whether a defendant is convicted, based on “standardized disposition” in CJARS adjudication table. *Incarceration sentence* captures whether a judge imposes a custodial or noncustodial sentence, based on “raw sentence from source” in the CJARS adjudication table. Non-custodial sentence, also known as community sentence, typically uses fine, community service, or probation term as alternatives to a prison sentence. Judges usually make two distinct decisions in sentencing convicted offenders, by first deciding whether to incarcerate the offender and then, once the incarceration decision has been made, determining the length of the prison sentence. Even though both incarceration sentence and prison term reflect the sentence severity, we will only use incarceration sentence here because judges might consider offenders’ neighborhood environment when imposing custodial or noncustodial sentence.

### Neighborhood-level variables

To study the neighborhood effects on CJ contact among focal cohorts, we will use tract-level variables for 786 census tracts in Harris County from public access IPUMS in 2000. For each tract, demographic variables, such as *total population*, *male population* (the percent of male), and *young population* (the percent of population aged 15 to 30) are included to reflect the gender and age that are most often associated with criminal offending (Elonheimo et al., 2014). *CJ population* in a tract will be calculated by aggregating focal cohorts in CJARS roster table based on their census tract identifiers. *CJ population rate* is then calculated by dividing CJ population by the total population in a corresponding tract.

All tract-level socioeconomic variables in 2000 are selected under the framework of social disadvantage theory. Social disorganization theory (Shaw & McKay, 1969), the most prominent theory on criminogenic neighborhood environment, emphasizes how structural factors, such as concentrated disadvantage, ethnic heterogeneity, and residential instability, affect the process of the informal social control and crime level in neighborhoods (Morenoff & Sampson, 1997). Concentrated disadvantage combines factors, *poverty* (the percent of people below the poverty line), *unemployment* (the percent of people 16 years old and over within the civilian labor force who are not employed), and *family disruption* (the percent of female-headed households with children out of all households with children younger than 18 years old). A combined measure of a racial diversity index and Latino and foreign-born concentration are used as indicative of ethnic heterogeneity. *Race diversity* index is calculated as 1 − ∑pi^2^, where pi is the proportion of a given ethnic group, which is squared and summed across the six race groups, White, Black or African American, American Indian or Alaska Native, Asian, and Native Hawaiian or Other Pacific Islander, and Some Other Race. This index reflects the probability that two randomly drawn individuals would differ in race (Osgood & Chambers, 2000). *Hispanic origin* is measured by the percent persons of Hispanic or Latino origin. *Immigrant concentration* is measured by the percent of foreign-born population. *Residential instability* is measured by the percent of individuals who moved residences within the previous 5 years.

To study CJ contact’s impacts on trajectories of neighborhood attainment among focal subcohorts, we need to examine the neighborhood characteristics of offenders’ origin and destination residences by using tract-level variables for 786 census tracts in Harris County from public access IPUMS data in 2000 and 2010. Three neighborhood variables will be used, including two demographic variables, *total population* and *CJ population*, and a socioeconomic variable, *poverty*. CJ population will be calculated by aggregating focal subcohorts in CJARS roster table based on their census tract identifiers. CJ population rate is then calculated again by dividing CJ population by the total population in a corresponding tract. *Poverty* is used to measure the socioeconomic status of offenders’ origin and destination neighborhoods respectively. All three variables will be collected in 2000 and 2010 through IPUMS.

**Table 1:** Summary of individual-, event-, and neighborhood-variables

Variable	Scale	Data	Time
**Individual-level variables** (CJARS Harris County)			
Gender	male; female	roster table	2001
Age	years	roster table	2001
Race	White, Black, Asian or Pacific Islander, American Indian or Alaska Native, Other	roster table	2001
Ethnicity	Hispanic; non-Hispanic	roster table	2001
**Event-level variables** (CJARS Harris County)			
Offense type	person, property, drug, DUI, public order, other	arrest table	2001–2009
Statutory severity	felony-level charge; misdemeanor-level charge	adjudication table	2001–2009
Charging characteristics	the number of charges pressed	linking roster, arrest, and adjudication tables through cjars_id	2001–2009
Criminal history	prior arrest; prior incarceration	arrest and adjudication tables	1978–2000
Initial plea	plea of guilty; plea of not guilty	adjudication table	2001–2009
Charge alteration	charge increase, decrease, or no change	linking arrest and adjudication tables	2001–2009
Case dismissal	dismissal; non-dismissal	adjudication table	2001–2009
Adjudication disposition	conviction; non-conviction	adjudication table	2001–2009
Incarceration sentence	custodial sentence; noncustodial sentence	adjudication table	2001–2009
**Neighborhood-level variables** (IPUMPS Harris County)			
Population	Total population in a tract	Total population table	2000, 2010
CJ population	Total population with a CJ contact in a tract	Linking CJARS and restricted decennial census	2000, 2010
Male population	The percent male population	Persons by sex table	2000
Young population	The percent of population aged between 15 and 29	Persons by age table	2000
Poverty	percent of people below the poverty line	Persons below poverty level in previous year table	2000, 2010
Unemployment	percent of people (16 years old and over) within the civilian labor force who are not employed	Persons 16 years and over by labor force and employment status table	2000
Family disruption	percent of female-headed households with children out of all households with children younger than 18 years old	Households by household type table	2000
Race diversity	A diversity index will be calculated based on 6 race categories	Persons by race table	2000
Hispanic origin	percent of Hispanic or Latino	Persons of Hispanic or Latino origin table	2000
Immigrant concentration	percent of foreign born	Persons by nativity table	2000
Residential instability	percent of individuals who moved residences within the previous 5 years	Population 5 years and over by residence in 1995 table	2000

## Model for cumulative disadvantage in the CJ system

We utilize individual-, neighborhood-, and event-variables to examine neighborhood effects on CJ contact. We will estimate the effects of offenders’ neighborhood conditions on their intermediate and final case outcomes, controlling for individual and case characteristics. The hypothesis is that offenders from more disadvantaged neighborhood will be more likely than similarly situated offenders with same individual and case characteristics to experience outcome-specific disadvantages at different stages of criminal case processing, and cumulative disadvantages across combinations of more punitive criminal case-processing outcomes (Kutateladze et al., 2014).

We will also estimate the interactions of some individual factors and case types because they may represent a source of disadvantage. Cumulative disadvantage may involve the interactive effects of specific constellations of individual defendant and case characteristics (Doerner & Demuth, 2010). Main effects of age, gender, and race can be magnified when examined in concert and along with certain case characteristics. Some individuals are treated more punitively because they possess specific combinations of characteristics. For example, young, male, minorities are singled out for the harshest punishments (Spohn & Holleran, 2000) and these inequalities can be even more pronounced when charged with drug or weapons offenses.

Our independent variables, neighborhood conditions, are at tract-level, including demographic variables, male population, young population, and CJ population, and socioeconomic variables, poverty, unemployment, family disruption, race diversity, Hispanic origin, immigrant concentration, and residential instability. These demographic and socioeconomic variables all reflect criminogenic neighborhood environments which can directly or indirectly associated with more punitive case outcomes.

Three groups of control variables at individual-level are used in the analysis. The first group of variables consists of extra-legal offender characteristics: age, sex, race, and ethnicity. The second group of variables are legally defined offender and offense characteristics: offense type, statutory severity, charging characteristics, and criminal history. The third group of variables involves procedural factors: initial plea and charge alteration. The effect of some offender characteristics on final disposition can be indirect, and mediated by legal categorizations. The seriousness of the initial charge, the number of charges, and prior arrest and incarceration records are all substantively and causally related to final disposition (Hagen, 1974).

Three dependent variables are the intermediate or final case outcomes, *case dismissal*, *adjudication disposition*, and *incarceration sentence*. To provide a comprehensive assessment of the criminal process, we consider the entire focal cohort, including offenders whose cases were dismissed, who were not convicted, and who were actually convicted and sentenced. Each criminal case progresses differently and can be terminated at different time points, data attrition is therefore expected when we follow all events in a CJ episode associated with focal cohorts longitudinally. For example, offenders who received dismissal decisions will not make it to adjudication and sentencing stages, hence have no record related to *adjudication disposition* or *incarceration sentence*. Considering the data attribution, we will first code *case dismissal* to include the entire focal cohorts, then code *adjudication disposition* to exclude the set of dismissed defendants, and finally code *incarceration sentence* to further exclude the set of defendants who received non-conviction.

To investigate neighborhood differences in outcome-specific and cumulative disadvantage,

We use two multivariate logistic regression models to investigate neighborhood effects on each CJ contact outcome, including case dismissal, adjudication disposition, and incarceration sentence. The first multivariate logistic regression models include the legal control variables (defendant characteristics, charging characteristics, statutory severity, offense type, prior record) and extra-legal control variables (age, sex, race, ethnicity). These exercises identify the parameters for an individual-level model that estimates the chance of being one type of case outcome and provide coefficients for the association between the set of control variables and each of the three dependent variables. They provide insight into baseline differences in case processing across demographic groups and legal case characteristics. The second and full models introduce neighborhood variables and incorporates all variables to estimate their effects on CJ contact. Multilevel logistic regression is used considering the hierarchical structure (individuals are nested in neighborhoods) in data. The general aim of multilevel logistic regression is to estimate the odds that a case outcome will occur while taking the dependency of data into account the fact that individuals are nested in neighborhoods. It will allow us to estimate such odds as a function of lower-level variables (e.g. individual’s race), higher level variables (e.g. neighborhood characteristics), and the way they are interrelated (cross-level interactions).

We will use lme4 pakage (version: 1.1–28) in R environment for multilevel logistic regression. Lme4 provides functions to fit and analyze linear mixed models, generalized linear mixed models and nonlinear mixed models using ‘Eigen’ and S4 classes (Bates et al., 2015). Glmer function in the package can fit a generalized linear mixed-effects model (glmm) and provide model estimates log-likelihood statistic via the Laplacian approximation.

The formal specification of logistic regression is:

$$\:P\left({Y}_{i}=1\right)=\:\frac{exp({B}_{0}+{B}_{1}*{X}_{i})}{1+exp({B}_{0}+{B}_{1}*{X}_{i})}$$

Is equivalent

$$\:Logit\left(odds\right)=\:{B}_{0}+{B}_{1}*{X}_{i}\:$$

The formal specification of multilevel logistic regression is:

$$\:Logit\left(odds\right)=\:{B}_{00}+\left(\:{B}_{10}+{\mu\:}_{1j}\:\right)*\:{X}_{i,j}+\:{\mu\:}_{0j}$$

j group for i observation,

$\:{B}_{00}$ is the fixed slope,

$\:{B}_{10}$ is the fixed slope for level 1 variable $\:{X}_{i,j}$

$\:{\mu\:}_{1j}\:$ is the deviation of the cluster-specific intercept from the fixed intercept

$\:{\mu\:}_{0j}\:$random intercept variance

## Model for CJ contact’s impact on neighborhood attainment

This subsection will investigate the inequality beyond the CJ system by estimating the impact of CJ contact on offenders’ neighborhood attainment. Different level of CJ contact may lead to various consequences on neighborhood attainment. Higher level contact or deeper involvement with the CJ system can lead to greater stigmatization and deeper detachment from social and economic institutions (Pager et al., 2009). This can exert a greater negative impact on offenders’ neighborhood attainment and make them to move to more geographically concentrated and economically disadvantaged neighborhoods.

We divide focal subcohorts into three groups to reflect different levels of CJ contact (Turney & Wakefield, 2019). This is achieved by summarizing offenders’ criminal outcomes documented in arrest and adjudication tables in CJARS Harris County between 2001 and 2009. The three groups are: (1) arrest-only offenders who were arrested in 2001 without further conviction (low level contact); (2) community-sentenced offenders who were arrested, adjudicated, and received a community sentence between 2001 and 2009 (middle level contact); (3) incarcerated offenders who were arrested, adjudicated, and incarcerated, but released from prison before the end of 2009 (high level contact). Among incarcerated offenders, level of CJ contact is further measured by a continuous variable, incarceration length, the number of months spent in prison. It may also explain disparities in neighborhood attainment, because longer prison sentences may further limit individuals’ choices of residential neighborhoods (Hipp et al., 2010). Incarceration length can be determined by the incarceration entry and exit date in CJARS Harris County incarceration Tables (2001–2009). Potential data attrition occurs when death, homelessness, or immigration, making offenders identified in decennial census 2000 untraceable in 2010.

In this part, we will examine three topical areas related to offenders’ neighborhood attainment, including spatial types of residential mobility, economic transition of neighborhood mobility, and the spatial concentration of the removed and returning CJ population among 786 census tracts in Harris County. In addition, we will investigate the relationship between the level of CJ contact and each of these three topical areas. Results provide evidence that CJ contact should be placed alongside structural and individual factors as an important predictor of offenders’ mobility among neighborhoods. Each analysis will be specified as below.

First, we will examine the spatial types of *origin-destination mobility* (Liu et al., 2018). The census tract is the neighborhood where people live and maintain social networks. Hence, five mutually exclusive spatial types of origin-destination mobility among subcohorts can be identified: no mobility (coded 0), within-tract mobility (coded 1), within-county inter-tract mobility (coded 2), within-state inter-county mobility (coded 3), and inter-state mobility (coded 4). No mobility is identified through comparing an offender’s MAFID in 2000 and that in 2010, which indicates whether the offender moved to different destination address from origin address (0 = same address; 1 = different address). All other four levels are identified based on the census tract identifier, GISJOIN, a 13-digit code concatenating 3-digit state code, 4-digit county code, and 6-digit tract code. By comparing an offender’s GISJOIN identifier in 2000 and that in 2010, we can identify this offender’s mobility pattern across tracts, counties, or states. We will examine whether there are distinguishable spatial types of mobility among subcohorts and provide descriptive statistics on the spatial types of mobility.

We can estimate the individual-level model to analyze how various factors are associated with the spatial types of mobility. The dependent variable is mobility type with five categories. Because the dependent variable has multiple categories, we adopt the multinomial logistic regression. The first category “no mobility” is used as the reference. Our independent variable is the level of CJ contact with three attributes, arrest-only (coded 0), community-sentenced (coded 1), and incarcerated (coded 2). Individual factors (age, sex, race, ethnicity) and tract-level variables (structural factors of social disorganization) of origin tracts are control variables. Multinomial logistic regression is used to examine the effect of the level of CJ contact and control variables on the odds of mobility types. This regression will provide coefficients for the association between the dependent and independent variables, as well as estimates of the characteristics of offenders’ mobility trajectories. We will know what factors push offenders away from their origin residences or neighborhoods, leading to relocations at various geographical settings. The mobility variable has a total of five categories (*J* = 5), which generates five different probabilities:

1 $$\:Pr\left({y}_{i}=1|{x}_{i}\right)={P}_{i1}=\frac{1}{1+exp\left({x}_{i}^{{\prime\:}}{\beta\:}_{2}\right)+exp\left({x}_{i}^{{\prime\:}}{\beta\:}_{3}\right)+exp\left({x}_{i}^{{\prime\:}}{\beta\:}_{4}\right)+exp\left({x}_{i}^{{\prime\:}}{\beta\:}_{5}\right)},$$

2 $$\:Pr\left({y}_{i}=2|{x}_{i}\right)={P}_{i2}=\frac{exp\left({x}_{i}^{{\prime\:}}{\beta\:}_{2}\right)}{1+exp\left({x}_{i}^{{\prime\:}}{\beta\:}_{2}\right)+exp\left({x}_{i}^{{\prime\:}}{\beta\:}_{3}\right)+exp\left({x}_{i}^{{\prime\:}}{\beta\:}_{4}\right)+exp\left({x}_{i}^{{\prime\:}}{\beta\:}_{5}\right)},$$

3 $$\:Pr\left({y}_{i}=3|{x}_{i}\right)={P}_{i3}=\frac{exp\left({x}_{i}^{{\prime\:}}{\beta\:}_{3}\right)}{1+exp\left({x}_{i}^{{\prime\:}}{\beta\:}_{2}\right)+exp\left({x}_{i}^{{\prime\:}}{\beta\:}_{3}\right)+exp\left({x}_{i}^{{\prime\:}}{\beta\:}_{4}\right)+exp\left({x}_{i}^{{\prime\:}}{\beta\:}_{5}\right)},$$

4 $$\:Pr\left({y}_{i}=4|{x}_{i}\right)={P}_{i4}=\frac{exp\left({x}_{i}^{{\prime\:}}{\beta\:}_{4}\right)}{1+exp\left({x}_{i}^{{\prime\:}}{\beta\:}_{2}\right)+exp\left({x}_{i}^{{\prime\:}}{\beta\:}_{3}\right)+exp\left({x}_{i}^{{\prime\:}}{\beta\:}_{4}\right)+exp\left({x}_{i}^{{\prime\:}}{\beta\:}_{5}\right)},$$

5 $$\:Pr\left({y}_{i}=5|{x}_{i}\right)={P}_{i5}=\frac{exp\left({x}_{i}^{{\prime\:}}{\beta\:}_{5}\right)}{1+exp\left({x}_{i}^{{\prime\:}}{\beta\:}_{2}\right)+exp\left({x}_{i}^{{\prime\:}}{\beta\:}_{3}\right)+exp\left({x}_{i}^{{\prime\:}}{\beta\:}_{4}\right)+exp\left({x}_{i}^{{\prime\:}}{\beta\:}_{5}\right)}.$$

In Eqs. 1, 2, 3, 4 and 5, *β*_2_, *β*_3_, *β*_4_, and *β*_5_ denote the specific effects of the independent variables for the second, third, fourth, and fifth categories, taking the first category (no mobility) as the reference. Note that the equation for *P*_*i*1_ derives from the fact that the five possibilities add to one [*P*_*i*1_=1–(*P*_*i*2_+*P*_*i*3_+*P*_*i4*_+*P*_*i5*_)]. The probabilities of response of the dependent variable depend on the nonlinear transformations of the linear function $\:{x}_{i}^{{\prime\:}}{\beta\:}_{j}={\sum\:}_{k=0}^{K}{\beta\:}_{jk}{x}_{ik}$, where *K* is the number of independent variables.

Second, we examine the strength and direction of offenders’ economic transition based on their neighborhood mobility. CJ contact may inhibit offenders’ upward neighborhood mobility and lead to downward neighborhood mobility. On one hand, for offenders previously living in disadvantaged neighborhoods, CJ contact experience will serve as a barrier to move up. On the other hand, individuals with CJ contact (or those with higher level CJ contact) will be more likely than those without CJ contact (or those with lower-level CJ contact) to move from nonpoor to poor neighborhoods (Warner, 2016).

We will adopt the cases of inter-tract mobility within Harris County by further narrowing down the sample to include only subcohorts whose *origin-destination mobility* equals to 2. Poverty rate is used to classify census tracts into two groups, poor (poverty rate above 20%) and nonpoor neighborhoods (poverty rate below 20%). The 20% poverty rate cut-off is consistent with previous treatments of neighborhood poverty (Wilson, 1987). We can identify each offender’s origin tract’s and destination tract’s economic status (poor or nonpoor) respectively. Contingency table based on two dimensions (poor/nonpoor and origin/destination) can summarize the economic transition of offenders. We will provide descriptive statistics of upward (poor to nonpoor), downward (nonpoor to poor), and lateral (poor to poor; nonpoor to nonpoor) mobility. Also, a description will be provided for economic transition based on offenders’ three levels of CJ contact.

We will then examine the association between offenders’ levels of CJ contact and their neighborhood economic transition. Economic transition will be used as the dependent variable. Each offender will be assigned a value indicating his or her extent and direction of economic transition. For example, if an offender moves from tract A in 2000 to tract B in 2010, the *economic transition* is calculated as (Poverty rate of B- Poverty rate of A)/Poverty rate of A. Both poverty rates of A and B will be based on public decennial census 2010. A negative (positive) number represents an upward (downward) neighborhood mobility, and a larger absolute number suggests a greater extent of economic transition. Independent variable is the level of CJ contact (arrest-only, community-sentenced, and incarcerated). Control variables include all individual variables (age, sex, race, ethnicity) and tract-level variables measuring social disadvantage of origin tracts. For example, research has found that although minorities tend to move into poorer neighborhoods than whites after a prison spell, this is mainly due to the residential segregation by race rather than the impact of incarceration itself (Massoglia et al., 2013). It will help us to understand, after controlling for individual characteristics and neighborhood conditions of origin tracts, the impact of CJ contact on offenders’ direction and extent of neighborhood economic transition.

Hierarchical Linear Modeling (HLM) will be used in modeling the association between the level of CJ contact and neighborhood economic transition. The brms package in R provides an interface to fit Bayesian generalized (non-)linear multivariate multilevel models using Stan. A wide range of distributions and link functions are supported, allowing users to fit linear, robust linear, count data, ordinal, and self-defined mixture models all in a multilevel context. In addition, all parameters of the response distribution can be predicted in order to perform distributional regression. Prior specifications are flexible and explicitly encourage users to apply prior distributions that actually reflect their beliefs. Model fit can easily be assessed and compared with posterior predictive checks and leave-one-out cross-validation.

Hierarchical linear model

$$\:{Y}_{ij}=\:{\beta\:}_{0j}+\:{\beta\:}_{1j}{X}_{ij}+\:{r}_{ij}$$

$\:{Y}_{\text{i}\text{j}}$ dependent variable measured for *i*th level-1 unit nested within the *j*th level-2 unit.

$\:{X}_{\text{i}\text{j}}$ value on the level-1 predictor

$\:{\beta\:}_{0j}$ intercept for the *j*th level-2 unit,

$\:{\beta\:}_{1j}$ regression coefficient associated with for the *j*th level-2 unit

$\:{r}_{ij}$ random error associated with the *i*th level-1 unit nested within the *j*th level-2 unit

Third, we will examine the spatial pattern of offenders’ neighborhood transitions among 786 census tracts in Harris County. It aims to understand the spatial pattern and regularity generated by offenders’ mobility among neighborhoods. We will examine the neighborhood patterns of subcohorts (mobility type = 2) in this part: offenders who were arrested from neighborhoods in Harris County in 2001, and then remained or reentered neighborhoods in Harris County some time before 2010. The CJ population rate in a tract is calculated by dividing the number of the CJ population by the total population in that tract. Comparing the spatial distribution of such rates among all 786 tracts between 2000 and 2010 can provide descriptive statistics regarding the spatial trend of CJ population over time. We can also provide measures of spatial disparity of CJ population removal rates in 2000 and share of returning CJ population in 2010 among tracts. We will use 2010 TIGER/Line + GIS data from public IPUMS NHGIS (https://data2.nhgis.org/main). NHGIS modified the TIGER/Line based on boundaries derived from the U.S. Census Bureau’s 2010 TIGER/Line files, and added a GISJOIN attribute field, which supplies standard identifiers that correspond to the GISJOIN identifiers in NHGIS data tables. OpenGeoDa is part of the Application system of RDC, and it can be used for this part of analysis (Anselin & McCann, 2009).

Exploratory Spatial Data Analysis (ESDA) can reveal complex spatial phenomenon not identified otherwise and is considered as a descriptive step before suggesting dynamic factors to explain the spatial patterns (Anselin, 2005). Under ESDA framework, the global and local autocorrelation can be used to analyze CJ population rates at two time points. Our outputs can suggest the existence of spatial interaction effects among neighborhoods. Global autocorrelation is assessed by global Moran’s I statistic. It measures spatial autocorrelation based on both feature locations and feature values simultaneously. Global Moran’s I of CJ population rates will be estimated at significant levels of 0.05 and 0.01. A positive and significant Moran’s I value indicates a general pattern of clustering in space of similar values (Anselin, 1995). Comparing global spatial dependence levels for all tracts at different time points can demonstrate the grow/decline of spatial clustering level of CJ population over time.

Similar global spatial clustering levels at different time points might mask a dramatic spatial restructuring. Compared with the global indicator, Local Indicator Spatial Autocorrelation (LISA) considers spatial proximity for each tract, which can help to identify significant local spatial clusters of CJ population rate, as well as analyze local spatial instability and spatial regimes (Anselin, 1995). A LISA significance map can be generated at each time point showing the tracts with significant LISA values and influential observations (hot spots) can thus be identified on the map. We suggest a temporal stability mapping of hot spots by overlaying hot spots in two time points for CJ population rate, which extends LISA significance map into a temporal context. More specifically, four categories of temporal hot spot stability are identified: a tract is labeled as “1, 1”, if this tract remains the hot spot of CJ population rate in both time points; a tract is labeled as “1, 0”, if it is a hot spot only in 2000; a tract is labeled as “0, 1”, if it is a hot spot only in 2010; a tract is labeled as “0, 0”, if it is never been a hot spot. Then, we can identify the total number of tracts that have been CJ population rate hot spot, and provide tract-level profiles for each of these four categories of tracts, such as the concentrated disadvantage, residential mobility, and racial heterogeneity.

Because the CJ population here includes three groups—arrest-only, community-sentenced, and incarcerated—we can conduct global and local spatial autocorrelation analyses separately for each group of offenders and then compare the spatial concentration changes across these groups. This approach allows us to determine whether the level of CJ contact impacts spatial concentration. For instance, prison sentences typically result in more stigma and deeper detachment from social and economic institutions. Consequently, people released from prison have very limited residential neighborhood choices, leading to their geographic concentration in certain neighborhoods. Therefore, incarcerated offenders’ residing neighborhoods are likely to be more geographically concentrated compared to those of offenders with only arrest records or community sentences.

In addition to gaining an understanding of the role of spatial dependence in the analysis of spatial patterns, we are also interested in the consistency of these effects over time (Rey & Ye, 2010).

The conditioned scatter plot can provide us an overall picture of the change of the CJ population rate over time (conditioned on years) with the spatial proximity considered. The scatterplot can contain 1,572 points (786 tracts at two time points) in two colors where a lighter color represents data in 2000, and the darker color represents those in 2010. For each point, the x-axis corresponds to the focal tract’s CJ population rate, and y-axis corresponds to its spatial lag which is computed using the average of the focal tract’s neighboring tracts’ CJ population rates. Considering spatial lag introduces spatial proximity into the analysis, which is crucial for computing spatial autocorrelation tests and specifying spatial regression models. If most of the lighter points concentrate in the lower left section of the scatterplot, while the darker dots scatter and locate in the upper part, it indicates that the CJ population rates rise over time at the tract level, along with the emergence of more diverse patterns. The scatterplot only shows a general pattern without identifying or labeling specific tracts to protect privacy.

##  Summary

This paper presents a research framework aimed at examining how neighborhood factors affect CJ contact and contribute to disparities across different stages of the justice system. It conceptualizes the punishment process as a series of dynamic decision points, focusing on the impact of neighborhood context on offenders’ CJ trajectories and post-CJ residential inequality. Using Harris County, Texas, as a case study, the framework assesses the combined influence of neighborhood characteristics, individual attributes, and event-specific variables on CJ outcomes. The study emphasizes the critical importance of understanding neighborhood mobility and its broader implications for community development and policy. Leveraging data from the Federal Statistical Research Data Centers and the Criminal Justice Administrative Records System, the analysis will offer an in-depth examination of offenders’ spatial patterns and economic transitions. The findings highlight the pivotal role neighborhood context plays in shaping CJ disparities and underscore the necessity of investigating neighborhood mobility among justice-involved individuals. This research will also support the Census Bureau’s mission to produce detailed crime and justice statistics and enhance the application of Census data for public policy.

In addition to examining how neighborhood factors influence CJ contact, this research holds broader implications for community development and public policy. Understanding the spatial mobility of justice-involved individuals can inform targeted interventions in neighborhoods disproportionately affected by CJ disparities (Campbell et al., 2020; DeMarco, 2024). By identifying patterns of residential instability, policymakers can allocate resources more effectively to support reentry programs, reduce recidivism, and address the root causes of neighborhood disadvantage (Drukker et al., 2005). Furthermore, this framework can guide the development of policies aimed at promoting social equity and improving community resilience, ultimately fostering safer and more stable neighborhoods (Turney & Wakefield, 2019).

However, as a conceptual paper, this study has limitations due to the lack of experimental validation. While the framework provides a comprehensive approach to understanding neighborhood effects on CJ contact, the absence of empirical testing and validation means that the proposed relationships and mechanisms have not yet been quantitatively assessed. Future research should empirically test and validate this framework to strengthen its applicability and robustness. Specifically, longitudinal studies could be conducted to follow offenders over extended periods, beyond the initial post-CJ contact phase, providing deeper insights into the long-term effects of neighborhood environments on reentry success and recidivism. Expanding the research to include various urban areas with diverse demographic and socioeconomic profiles would also allow for comparative analysis of neighborhood effects on CJ contact and disparities across different contexts.

Further, evaluating the impact of specific policy interventions aimed at alleviating neighborhood disadvantages and supporting reentry programs could help identify effective strategies for reducing CJ contact disparities. Incorporating additional data sources, such as employment records, educational backgrounds, and health information, would enable a more comprehensive analysis of the socioeconomic factors influencing CJ contact and mobility patterns. Complementing quantitative findings with qualitative methods, such as interviews and focus groups with justice-involved individuals, would provide deeper insights into the personal and contextual factors affecting their experiences and mobility trajectories. Pursuing these future research directions will not only empirically validate the proposed framework but also provide a more nuanced understanding of the intricate relationship between neighborhood factors and criminal justice outcomes, ultimately leading to more effective and informed policymaking.

## Data Availability

Not applicable.
